# Sunflower seed cake as a source of nutrients in gluten-free bread

**DOI:** 10.1038/s41598-023-38094-w

**Published:** 2023-07-05

**Authors:** Agata Blicharz-Kania, Anna Pecyna, Beata Zdybel, Dariusz Andrejko, Andrzej Marczuk

**Affiliations:** 1 Department of Biological Bases of Food and Feed Technologies, University of Life Sciences in Lublin, Głęboka 28, 20-612 Lublin, Poland; 2 Department of Technology Fundamentals, University of Life Sciences in Lublin, Głęboka 28, 20-612 Lublin, Poland; 3 Department of Agricultural Forestry and Transport Machines, Faculty of Production Engineering, University of Life Sciences in Lublin, Głęboka 28, 20-612 Lublin, Poland

**Keywords:** Mechanical engineering, Nutrition

## Abstract

An increase in the demand for cold-pressed vegetable oils can be observed, e.g. from sunflower. The press cake formed during sunflower oil production can also be an important source of protein, carbohydrates, and phenolic compounds. The aim of the study was to examine the quality of gluten-free breads fortified with sunflower seed cake. The fortified products were characterized by lower moisture content (49.35–48.87%). The bake loss parameter decreased after the use of the highest 15% dose of the sunflower cake. The addition of the sunflower cake caused an increase in the content of nutrients, compared to the control sample: protein (7.44–9.69%_d.b._), fat (3.41–10.72%_d.b._), crude fiber (1.23–2.34%_d.b._), polyphenols (89.3–222.3 mg·100 g_d.b._^−1^), and soluble sugars (2.42–2.73%_d.b._). The gluten-free breads with the sunflower seed cake exhibited lower hardness, springiness, and chewiness but higher cohesiveness. The use of the additive contributed to the darkening of the gluten-free bread crumb. The appearance, consistency, aroma, and palatability of the sunflower cake-fortified gluten-free bread were found to be much more attractive than the parameters of the unmodified bread. The conducted research has shown that, thanks to sunflower cake addition, it is possible to obtain a highly nutritious product with desirable sensory quality.

## Introduction

Celiac disease is one of the most common food intolerances. The number of patients is constantly growing and the disease has been estimated to affect approximately 1% of the global population^1^. In genetically predisposed people, the disease is a consequence of gluten consumption. It should be noted, however, that celiac disease is not the only condition caused by gluten intake. The general term “gluten-related disorders” refers to a substantially higher number of diseases, e.g. hypersensitivity or allergy to gluten. The number of subjects who must use a gluten-free diet as part of the treatment of these diseases is still on the increase^2,3^.

Gluten-free bread is the basic product consumed by subjects suffering from the aforementioned disorders. Despite the growing researchers’ interest in improvement of the quality of gluten-free bread, consumers are still dissatisfied with the quality of this product^3,4^. Gluten-free bread often fails to have desirable technical and functional properties or nutritional values^2,5^. It has lower content of active ingredients than classic cereal products. Most frequently, it is characterized by substantially lower levels of protein, minerals, vitamins, and dietary fiber but higher content of available carbohydrates and fat^5,6^. It is therefore advisable to enrich bread with oilseeds containing nutritionally valuable micro- and macroelements, vitamins, essential fatty acids (EFA), and antioxidants (tocopherols, polyphenolic compounds, carotenoids). Oilseeds increase the nutritional value of bakery products and can exert a positive effect on their sensory attractiveness^1,7^.

With their favorable chemical composition, sunflower seeds are recommended to be introduced into the diet. It is assumed that 100 g of this raw material contain 20.78 g of protein, 51.46 g of fat, 20 g of carbohydrates, 3.02 g of ash, and 8.6 g of fiber. Sunflower seeds are also a valuable source of minerals, as they contain 78 mg of calcium, 5.25 mg of iron, 645 mg of potassium, 5 mg of zinc, 9 mg of sodium, 325 mg of magnesium, 660 mg of phosphorus, 1.89 mg of copper and 53 µg of selenium per 100 g. In addition, caffeic, chlorogenic, and ferulic acids, i.e. polyphenols present in sunflower seeds, have high antioxidant potential. The total content of phenolic compounds in sunflower seeds is 2700 mg 100 g^−1^ on a dry weight basis. The material is also a source of essential fatty acids (EFA), e.g. oleic and linoleic acid^8–10^. These compounds exert an impact on the function the nervous, cardiovascular and brain systems, and the healthy appearance of nails, skin, hair. They have an antidepressant effect and reduce the symptoms associated with premenstrual tension^11^.

By-products obtained during oil production may be an important source of protein, minerals, and polyphenols. They also contain some vitamins and fat remaining after the pressing process as well as large amounts of micro- and macroelements^12–14^. It can be assumed that enrichment of gluten-free bread with oilseed cake may improve its health values and consumer attractiveness^15^. The novelty in our research was the use of unprocessed sunflower cake from the cold pressing process as an additive intended for consumers with gluten intolerance. The cold pressing process is the least invasive method for oil pressing is carried out at temperatures up to 50 °C and does not generate waste containing synthetic solvents. The by-products obtained in this way are additionally characterized by relatively high content of antioxidants, fatty acids, minerals, vitamins, and natural pigments.

The aim of the study was to assess the impact of the use of sunflower cake on the nutritional value and selected quality parameters of gluten-free bread (Table 1).

**Table 1:** Model of experiment parameters of gluten-free bread with sunflower seed cake.

Ingredients	SC0	SC5	SC10	SC15
Rice flour (g)	250	225	200	175
Sunflower seed cake (g)	0	25	50	75
Corn flour (g)	200	200	200	200
Potato starch (g)	50	50	50	50
Water (cm^3^)	500	500	500	500
Rapeseed oil (g)	30	30	30	30
Dry yest (g)	8	8	8	8
Salt (g)	8	8	8	8
Sugar (g)	2.5	2.5	2.5	2.5
Ground flax seeds (g)	15	15	15	15

## Results

### Physical properties of bread

The physical parameters of the tested breads calculated based on the measurements are presented in Table 2. The increase in the amount of the sunflower cake was accompanied by a decline in the moisture content of the product. However, there were no statistically significant differences between samples SC0 and SC5. The mean moisture of the crumb of the analyzed breads ranged from 48.64 to 49.37%.

**Table 2:** Physical properties of gluten-free bread with sunflower seed cake.

Probe	Crumb moisture (g·100 g^−1^)	Bread volume (cm^3^)	Total baking loss (g_loss_·100 g^−1^)
SC0	49.35 ± 0.02^c^	1256.64 ± 50.73^a^	12.42 ± 0.11^a^
SC5	49.37 ± 0.01^c^	1223.04 ± 30.79^a^	12.36 ± 0.06^a^
SC10	48.64 ± 0.05^b^	1182.72 ± 11.63^a^	12.00 ± 0.14^b^
SC15	48.87 ± 0.01^a^	1182.72 ± 11.38^a^	11.97 ± 0.01^b^

SC0—control probe, SC5—gluten-free bread with 5% sunflower cake added; SC10—gluten-free bread with 10% sunflower cake added; SC15—gluten-free bread with 15% sunflower cake added. Data are presented as mean (n = 3) ± standard deviation, letters ^a, b, c^ indicate homogeneous groups in Tukey's post-hoc test (*p*=0.05).

**Figure 1:**
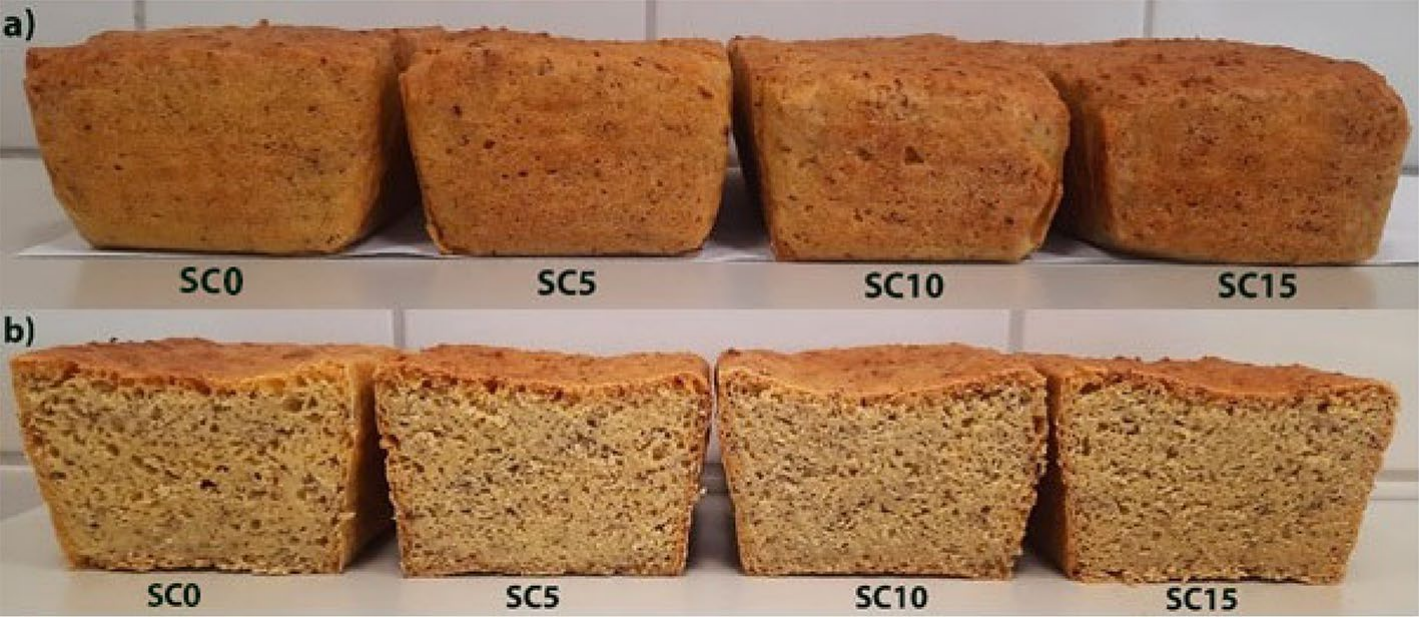
External appearance (**a**) and cross-section (**b**) of bread with different sunflower cake content: SC0—control probe; SC5—gluten-free bread with 5% sunflower cake added; SC10—gluten-free bread with 10% sunflower cake added; SC15—gluten-free bread with 15% sunflower cake added.

The volume of the sunflower cake-supplemented gluten-free bread decreased with the increase in the percentage of the by-product (Fig. 1). Noteworthy, the decrease in the volume was not statistically significant (*p* = 0.051).

The modification of the gluten-free bread recipe resulted in significant changes in bake loss. The highest values of this parameter were recorded in the control bread, and the losses were comparable (*p* > 0.05) to those observed in the sample enriched with 5% of the by-product.

### Chemical composition

The addition of the sunflower seed cake, SC to the gluten-free bread recipe caused significant changes (*p* < 0.05) in all the analyzed nutrients (Table 3). The increase in the dose of the sunflower seed cake was accompanied by an increase in the content of protein, fat, and crude fiber in the gluten-free breads. The statistical analysis revealed significant changes in each variant with the 5% increase in the dose of the additive.

**Table 3:** Chemical compositions of gluten-free bread with sunflower seed cake.

Probe	Protein (%_d.b._)	Fat (%_d.b._)	Soluble sugars (%_d.b._)	Polyphenols (mg·100 g_d.b._^−1^)	Crude fiber (%_d.b._)
SC0	7.44 ± 0.01^a^	3.42 ± 0.09^a^	2.42 ± 0.04^a^	89.30 ± 3.55^a^	1.230 ± 0.020^a^
SC5	8.52 ± 0.02^b^	6.58 ± 0.12^b^	2.53 ± 0.06^ab^	148.70 ± 0.65^b^	1.920 ± 0.017^b^
SC10	9.31 ± 0.02^c^	7.79 ± 0.16^c^	2.62 ± 0.04^bc^	190.60 ± 0.75^c^	1.993 ± 0.015^c^
SC15	9.69 ± 0.04^d^	10.72 ± 0.02^d^	2.73 ± 0.09^c^	222.30 ± 6.03^d^	2.343 ± 0.031^d^

SC0—control probe, SC5—gluten-free bread with 5% sunflower cake added; SC10—gluten-free bread with 10% sunflower cake added; SC15—gluten-free bread with 15% sunflower cake added. Data are presented as mean (n = 3) ± standard deviation, letters ^a, b, c, d^ indicate homogeneous groups in Tukey's post-hoc test (*p*=0.05).

The sunflower cake supplementation also increased the content of soluble sugars. However, no significant changes were observed in the enrichment variant with 5% of sunflower cake (compared to sample SC0) and after the increase in the by-product dose from 10 to 15%.

The content of polyphenols in the control gluten-free bread was 89.30 mg·100 g^−1^. The fortified products contained up to 222.33 mg·100 g^−1^ of polyphenols in dry matter. The statistical analysis revealed significant changes in the content of these components depending on the additive dose used in the recipe.

### Textural properties

Hardness, defined as the force required for deformation of the product, is one of the most frequently defined mechanical parameters of bakery products. As shown by the statistical analysis, the hardness of the gluten-free bread decreased significantly with the increase in the sunflower cake dose (Table 4). The mean values of hardness determined 48 h after baking increased in all the samples. The results of the statistical analysis proved the significance of these differences. Additionally, during storage, the greatest changes in the hardness parameter were noted in the 5% SC-supplemented bread (68.4% of the value determined 24 h after baking).

**Table 4:** Texture profile analysis of gluten-free bread with sunflower seed cake.

Storage time (h)	Probe	Hardness (N)	Elasticity (−)	Chewing (N)	Cohesiveness (−)
24	SC0	42.62 ± 2.40^c^	0.968 ± 0.008^d^	7.18 ± 0.75^de^	0.174 ± 0.017^a^
	SC5	33.24 ± 2.16^b^	0.792 ± 0.076^c^	5.12 ± 0.31^b^	0.196 ± 0.018^ab^
	SC10	34.92 ± 2.43^b^	0.576 ± 0.048^b^	4.35 ± 0.53^ab^	0.218 ± 0.033^abc^
	SC15	25.16 ± 2.59^a^	0.560 ± 0.046^b^	3.23 ± 0.64^a^	0.230 ± 0.037^bcd^
48	SC0	59.34 ± 5.77^d^	0.738 ± 0.016^c^	8.21 ± 0.63^ef^	0.188 ± 0.011^ab^
	SC5	55.98 ± 3.26^d^	0.612 ± 0.061^b^	8.57 ± 0.51^f^.	0.252 ± 0.022^cde^
	SC10	43.66 ± 4.70^c^	0.550 ± 0.014^ab^	6.50 ± 0.37^ cd^	0.272 ± 0.018^de^
	SC15	38.40 ± 3.76^bc^	0.486 ± 0.013^a^	5.31 ± 0.83^bc^	0.294 ± 0.015^e^

SC0—control probe, SC5—gluten-free bread with 5% sunflower cake added; SC10—gluten-free bread with 10% sunflower cake added; SC15—gluten-free bread with 15% sunflower cake added. Data are presented as mean (n = 5) ± standard deviation, letters ^a, b, c, d, e, f^ indicate homogeneous groups in Tukey's post-hoc test (*p*=0.05).

The statistical analysis also confirmed the significance of the changes in bread springiness induced by the addition of the sunflower seed cake, as the mean values of the parameter decreased after the enrichment. It should be noted, however, that the increase in the dose of the by-product from 10 to 15% in the present study did not exert a significant effect on the springiness of the fresh bread and samples analyzed after 48 h. The smallest differences in springiness were noted in samples SC10 and SC15 stored for 48 h.

The mean values of chewiness of the tested bread (determined 24 h after baking) were in the range of 7.18–3.23 N (control and sample SC15, respectively). Compared to sample SC10, the value of this parameter in the bread samples analyzed after 24 and 48 h did not change significantly after the increase in the sunflower cake dose by another 5%. The mean values of chewiness increased significantly during storage (up to 8.57 N) in the sunflower cake-enriched bread (regardless of the content of the additive). As shown by the statistical analysis, the chewiness of sample SC0 did not change significantly after 48 h of storage.

The addition of 5 and 10% of the sunflower seed cake did not cause significant changes in the cohesiveness of the gluten-free bread. In contrast, the product fortified with 15% of SC exhibited significantly higher cohesiveness. The mean cohesiveness values increased during storage but, in comparison with the control sample, these changes were not significant. The results of the statistical analysis indicated that the cohesiveness of the sunflower cake-enriched products (regardless of the additive dose) did not differ significantly in the bread samples stored for 48 h.

### Color of bread

In addition to taste and texture, color is an important sensory indicator of baked goods and an important factor in the meeting consumer expectations. Bread color depends on the technological parameters during baking, the recipe, and the characteristics of the dough. Table 5 presents the results of the analysis of the color of gluten-free bread supplemented with 5%, 10%, and 15% of sunflower seed cake and the control sample. The use of the sunflower cake additive induced changes in the color parameters (L*, a*, b*, and C*).

**Table 5:** Color parameters of gluten-free bread supplemented with sunflower seed cake.

Probe	L*	a*	b*	C*	Δ*E*
SC0	61.87 ± 0.89^c^	1.72 ± 0.23^a^	22.86 ± 0.88^a^	22.93 ± 0.88^a^	
SC5	59.46 ± 1.39^b^	2.16 ± 0.33^b^	23.65 ± 0.96^ab^	23.75 ± 0.95^ab^	2.58
SC10	58.96 ± 0.96^b^	2.27 ± 0.19^b^	24.18 ± 1.34^bc^	24.29 ± 1.35^bc^	3.25
SC15	55.37 ± 2.41^a^	2.48 ± 0.29^b^	25.09 ± 0.87^c^	25.21 ± 0.86^c^	6.91

SC0—control probe, SC5—gluten-free bread with 5% sunflower cake added; SC10—gluten-free bread with 10% sunflower cake added; SC15—gluten-free bread with 15% sunflower cake added. Data are presented as mean (n = 10) ± standard deviation, data value of each parameter with different superscript letter in rows are significantly different (Tukey test. *p* ≤ 0.05).

It was shown that the value of parameter L*, i.e. the lightness of the crumb, decreased with the increase in the sunflower cake dose. Noteworthy, the increase in the dose of the by-product from 5 to 10% did not cause statistically significant differences in this parameter. Bread SC15 was characterized by the lowest lightness value, probably due to the presence of a dark pigment in the sunflower cake (values of sunflower cake color parameters: L* = 73.11, a* = 2.18, b* = 9.32).

The values of parameters a* and b* increased with the increasing level of sunflower cake supplementation of the gluten-free bread. The addition of the cake to the dough caused an increase in the value of parameter a* (differences between samples SC0 and SC5), i.e. the intensity of redness increased. However, there were no statistically significant differences in parameter a* between samples enriched with 5 and 15% of the sunflower seed cake. The value of parameter b*, indicating chromaticity towards yellow (+ b*), increased and was the highest in sample SC15. Noteworthy, the 5% dose of the by-product in the bread did not exert a significant effect on parameter b*, compared to the control sample. Similar to the other parameters (a* and b*), parameter C* reflecting color saturation increased with the increasing sunflower cake doses. Nevertheless, the 5% dose of the by-product added to the gluten-free bread samples did not have a significant effect on color saturation expressed by parameter C*.

The criterion of absolute color difference (Δ*E*) was used in the analysis of the results as well. It indicates differences between the color of two samples: the greater the value, the greater the difference (the Δ*E* value was calculated relative to the color of the control sample). The value of this parameter in the bread samples enriched with 5% and 10% of the sunflower cake was in the range of 2 < Δ*E* < 3.5, indicating that the color difference was recognizable even to an inexperienced observer. However, the value of Δ*E* in the case of sample SC15 was higher than 5, which indicated a significant color change (the observer can clearly recognize two different colors).

### Sensory evaluation

**Figure 2:**
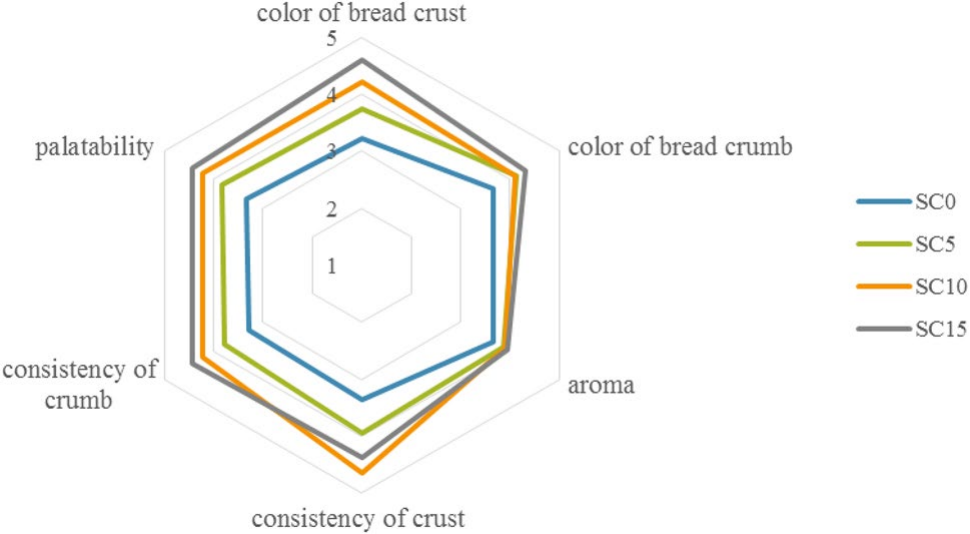
Sensory evaluation of gluten-free bread with the addition of sunflower cake: SC0—control probe; SC5—gluten-free bread with 5% sunflower cake added; SC10—gluten-free bread with 10% sunflower cake added; SC15—gluten-free bread with 15% sunflower cake added.

The results of the sensory evaluation are presented in Fig. 2. The best scores for the overall appearance were achieved by the bread enriched with 15% of the sunflower cake. As assessed by the panelists, the sunflower cake addition had a positive effect on the crust color change. The lowest score was given to the SC0 non-supplemented product (3.2) and the highest score (4.6) was achieved by the bread fortified with 15% of the sunflower cake. The color of the bread crumb received varied scores. The lowest (3.7) and highest (4.3) scores were given to the control sample and the product with 15% of the sunflower cake, respectively. It was clearly shown that the increasing sunflower cake doses were associated with higher scores for the color of the bread crust and crumb. Therefore, it can be concluded that the application of the higher doses of the additive in gluten-free bread recipes had a positive effect on its appearance. The results of the color assessment clearly indicate significant darkening of the crumb of the SC-enriched gluten-free breads in comparison with the control sample.

The palatability and aroma of a product are the most important organoleptic features exerting the greatest effect on consumer acceptance. In comparison with the control bread, the sunflower seed cake added to the dough mixture improved the scores of the palatability and aroma of the gluten-free bread samples. The data presented in Fig. 2 indicate that the addition of the sunflower cake only slightly improved the aroma of the gluten-free bread. However, the further increase in the sunflower cake dose to 15% did not have an impact on the attractiveness of the product aroma. The sunflower cake addition improved the taste of the gluten-free bread, as the palatability of this material was generally assessed positively. The lowest palatability score (3.3) was given to the cake non-supplemented bread. As assessed by the panelists, the palatability of the gluten-free bread increased with the increase in the sunflower cake dose.

The sunflower cake supplementation had a positive effect on the consistency of the material. The panelists gave the highest score (4.4) for the appropriate consistency of the crust to sample SC10, while the highest score for the consistency of the crumb was achieved by sample SC15 (4.4). The control bread sample received unfavorable consistency scores.

## Discussion

The experiment showed that not all physical properties of gluten-free bread changed significantly after the addition of 15% SC, e.g. the bread volume. As found by Lazaridou et al.^16^, additives used in the recipe for gluten-free bread have an impact on its volume. However, the amount of the ingredient is highly important. For example, Ziemichód & Różyło^17^ reported that the volume of gluten-free bread enriched with goji berries at levels that did not exceed 12% did not change significantly. In a study conducted by Shchekoldina & Aider^18^, where wheat bread was fortified with sunflower meal protein isolate (SMPI), no significant changes in the bread volume were found at 12% supplementation with this additive (with thick sourdough starter). The volume of gluten-free bread also depends on the type of flour used. In earlier studies^19^, rice bread had a significantly larger volume than corn bread, and intermediate volume values were found in bread made from mixtures of these flours with buckwheat flour. Therefore, the properties of the basic ingredients used in the recipe may be of paramount importance. In the case of gluten-free bread, the properties of the porous spatial structure of the crumb and hence the bread volume are associated with the presence of gas bubbles trapped in the porous crumb skeleton composed mainly of denatured proteins, gelatinized starch, and functional additives^20^.

On the other hand, the bread crumb moisture and the total baking loss decreased significantly after the addition of SC. Similar moisture values were reported by other authors analyzing gluten-free breads with a modified recipe^21–24^. As shown by literature data^25^, total bake loss in traditional mixed breads from dough weighing e.g. 600 g (mold bread) should range from approximately 13.3–16.7%. Noteworthy, the larger the dough aliquots are, the lower the bake loss is. The bake loss in gluten-free bread may range from 10 to even 22%, depending on the ingredients used^1,21,22,26^.

The improvement in the nutritional value of the modified products was associated with the significantly higher content of protein, fat, and fiber in the sunflower seed cake than in the rice flour^9,27–29^. Sunflower seeds are also a valuable source of antioxidants^9,15^. For example, the total phenolic content in raw seeds is approximately 332 mg·100 g^−1^^30^. Therefore, fortification of cereal products increases the content of polyphenols in the material^14,31,32^.

The gluten-free breads supplemented with the sunflower seed cake exhibited lower hardness, springiness, and chewiness as well as higher cohesiveness. The use of oilseeds in bread often has a negative effect on the hardness of the final product^1^. However, raw materials obtained from sunflower seeds may reduce the hardness of flour products. Similar results were reported by Zaky et al.^33^. The authors produced pasta enriched with sunflower meal protein isolate. It was found that this supplementation in the recipe contributed to the reduction in the hardness of both raw and cooked pasta.

In turn, other authors reported an unfavorable effect of oilseed cake supplementation on bread springiness^34,35^. Similar trends were observed in our research. Moreover, during storage, the springiness of the bread declined, which was associated with staling, i.e. aging of the bread. In general, the structure of stored bread undergoes many changes, i.e. loss of crumb springiness increasing at a variable rate^36^.

The gluten-free bread fortified with 15% of SC exhibited significantly higher cohesiveness. This may have been related to the strengthening of the dough structure. The proportions of ingredients in bread mixtures and the nature and molecular weight of individual proteins and saccharides have an impact on the properties of dough^37^. The mean cohesiveness values increased during storage, but these changes were not significant relative to the control sample. The cohesiveness of the sunflower cake-enriched products (regardless of the additive dose) did not differ significantly in the bread samples stored for 48 h. Similar trends in changes in chewiness and cohesiveness were reported by Salah et al.^38^. The authors enriched bread with gluten-free rapeseed protein, and the products were characterized by lower hardness and chewiness, with a slight increase in cohesiveness and springiness. As suggested by Salah et al.^38^, these changes can be explained by the involvement of rapeseed proteins in the formation of a protein network leading to the generation of intra- and intermolecular disulfide bonds (covalent bonds) linking pulp components, thus providing a better developed structure.

The use of the additive caused darkening of the gluten-free bread crumb, probably due to the presence of a dark pigment in the sunflower cake. As suggested by Koletta et al.^39^, the color of the crumb is usually similar to the color of additives, as the temperature in the inner part of bread is lower and exerts no significant impact on the color change.

The fortification caused an increase in parameters a*, b*, and C*. The same tendency of changes in crumb color parameters was described in the analyses of gluten-free bread supplemented with sunflower protein concentrate (SPC). As reported by Zorzi et al.^26^, SPC, which is a high-protein raw material, was responsible for the darker color of low-protein gluten-free bread. The color of the bread exhibited chromaticity towards red (+ a*), which increased with the higher dose of SPC. As suggested by the authors, the SPC-containing bread had lower crust and crumb lightness due to the dark color of the SPC additive. Noteworthy, darker products may be positively associated by consumers with wholegrain bread. Similar results were reported by Zaky et al.^33^, who used sunflower meal protein isolate (SMPI) for production of pasta. Their results showed darkening of the pasta color (L*, a*, and b*) with the increasing doses of SMPI.

Similarly, a study of gluten-free breads supplemented with sour cherry cake^40^ demonstrated substantially greater color differences caused by the additive in comparison with the control bread. Greater changes were observed in samples enriched with higher doses of the additive, which was associated with the color of the sour cherry cake.

Grasso et al.^41^ used defatted sunflower seed flour (DSSF) as a substitute for wheat flour for baking biscuits.The color of the DSSF-fortified biscuits was significantly darker than that of wheat flour biscuits. Similarly, the redness and blueness values were higher, and the DSSF-supplemented biscuits were browner than the control. Hussein et al.^42^ analyzed fortification of fino bread with sunflower meal (SFM), which resulted in a dark brown color of the product, compared to the control sample. In turn, the crumb of the SFM-supplemented bread had lower values of parameters a* and b* than the control bread.

The results of the color assessment clearly indicate significant darkening of the crumb of the SC-enriched gluten-free breads in comparison with the control sample. As suggested by Gumul et al.^40^, consumers may positively associate darker products with wholegrain bread.

The palatability of the gluten-free bread increased with the increase in the sunflower cake dose. This may have been related to the higher content of fat and soluble sugars in the samples fortified with the increasing SC levels. Fat is a carrier of many biologically active and aromatic substances. Sufficiently high fat content has a positive effect on the appearance, palatability, and stability of products^43^. Rice flour (which was replaced by sunflower cake) contains approximately 0.2–1.3% fat^44,45^; the flour used in our study contained 1% of fat. In turn, sunflower cake from cold pressing may still contain up to 31.4% of fat^13^. The sunflower cake used in the experiment contained 28% of fat.

In a study conducted by Grasso et al.^41^, the addition of DC (de-oiled sunflower cake) at different concentrations (18 and 36%) to a sponge cake recipe instead of wheat flour resulted in an increase in the protein content, total phenolic content, and antioxidant activity and enhanced the hardness and brownish color of the cake without altering its calorific value. In turn, Grasso, Liu and Methven^46^ showed that the addition of different concentrations (15% and 30%) of DC to muffin batter contributed to a dark brown color of the cupcakes, and better sensory quality was achieved in the 15% DC variant than at 30% supplementation.

As shown by literature data, the effect of supplementation of bakery products with de-oiled sunflower cake (DC) was similar^47^. Shchekoldina and Aider^18^ obtained bread with improved sensory quality after addition of 10% DC to plain flour. Other studies conducted by Bhise et al.^48^ demonstrated that biscuits baked with the use of plain flour supplemented with 10% of DC exhibited higher content of digestible protein, better technological properties, and good consumer acceptance. Similarly, other by-products, i.e. apple cake or strawberry seed cake, added to the recipe^6,49^ had a positive impact on the sensory quality of gluten-free bread.

## Summary

The results of the present experiment confirm the potential of by-products of the oil industry to be used as a raw material for the production of gluten-free bread. Noteworthy, the sunflower seed cake-fortified breads contained higher amounts of pro-health compounds and had better sensory quality. The fortified products were characterized by slightly lower moisture and volume. Additionally, the bake loss declined in the variant with the highest 15% dose of the sunflower seed cake. The sunflower cake addition increased the nutritional value of the products. The modification of the recipe contributed to a significant increase in the content of protein, soluble sugars, crude fiber, and fat. The sunflower cake fortification also increased the content of antioxidant compounds. Significant changes in the textural properties of the analyzed material were observed as well. The fortified gluten-free bread samples were characterized by lower hardness, springiness, and chewiness but higher cohesiveness. The color of the modified products changed significantly. The sunflower cake addition resulted in darkening of the gluten-free bread crumb and an increase in parameters a*, b*, and C*. These changes were positively assessed by the consumers. The SC-fortified gluten-free bread was found to be much more attractive in terms of its appearance, texture, taste, and aroma. The results indicate that sample SC15, i.e. gluten-free bread fortified with 15% of sunflower cake, had the most favorable sensory values.

The bread made according to the analyzed recipe is recommended for industrial production. Its texture and sensory properties differed significantly from those of the unmodified sample, but it was more attractive to consumers and contained valuable polyphenols and large amounts of protein and crude fiber. As shown in the present study, an appropriate selection of recipe ingredients may yield a highly nutritious product with desirable sensory quality.

In further analyses, it is advisable to modify the composition of gluten-free bread enriched with sunflower cake in order to improve the aroma. It would probably be possible to use coconut or almond cake for this purpose. It is also worth introducing by-products with different degrees of fragmentation to the bread, thus improving their structural properties.

## Material and methods

### Material

The study material was gluten-free bread fortified with sunflower seed cake at the level of 5, 10, and 15%, of the total amount of rice flour. The seeds for experiments were obtained from a local seed company (BRAT.pl sp. z o.o. sp. k. ul. Kilińskiego 66A 34-700 Rabka-Zdrój). The collection of plant material and experiment had been conducted in compliance with relevant guidelines and regulations. The by-products were obtained via the process of cold pressing of sunflower seeds with a screw press (Sana, EUJ- 702). The cake was ground in a laboratory knife mill (Chemland, FW100). Bread prepared with an unmodified recipe, i.e. rice flour, corn flour, potato starch, ground flax seeds, sugar, salt, and dry yeast, served as a control sample.

### Preparation of dough and bread baking procedure

The product was made and coded as indicated by the data in Table 1. The bread dough was prepared in a laboratory spiral mixer (Kenwood). The ingredients were combined by 5-min mixing. A 1030-g portion of the dough was placed in a mold and post-fermented for 40 min at 37 °C and 80% relative humidity. The breads were baked at 230 °C for 40 min in a convection-steam oven (Houno, DK 8940 Randers).

### Determination physical properties of bread

Moisture was determined with the gravimetric method. Approximately 2-g samples were dried until no loss of weight was observed. The process was carried out at a temperature of 130 °C using a laboratory dryer (POL-EKO, SLN 15 STD). Bread volume was measured with the rapeseed displacement method. Bread moisture was determined in accordance with the adopted method^50,51^. The total bake loss X [%] was calculated using the formula:* X* = ((*a*−*b*)·100)/*a,* where a—weight of dough before baking, b—weight of chilled bread^34^.

### Analysis of protein content

A Kjeltec apparatus (TM8400) and ASN 3100 software were used to determine the protein content. Distillation was carried out in an automated Kjeltec Auto device (Tecator). The nitrogen content was converted to protein using the conversion factor N × 6.25^51^.

### Determination of fat content

Total fat was determined via continuous ether extraction in a Soxtec device (TM8000). The AN 310 software was used to analyze the results.

### Determination of sugar content

The content of soluble sugars was determined with the HPLC technique in accordance with the PN 12,630:2002^52^ standard.

### Determination of fiber content

The content of crude fiber was determined by sequential extraction with hot H_2_S0_4_ (conc. 1.25%) and hot NaOH (conc. 1.25%). Rinsed, drained, and acetone-washed crucibles with the residual material were dried and the sample was incinerated. Its weight was determined after cooling. The percentage of crude fiber was calculated as the difference between the weight of the residue and ash in the total weight of the sample^50^.

### Determination of polyphenol content

A Cary spectrophotometer (100 Varian) was used to determine polyphenols. The analysis consisted in methanol extraction of active substances present in the material and measurement of the absorbance of solutions at a specific wavelength (relative to the zero sample)^53^. The content of total polyphenols was expressed as caffeic acid equivalents.

### Determination of textural properties

The bread crumb texture was analyzed on days 1 and 2 after baking. A testing machine (Zwick/Roell, Z0.5) with testXpert II software was used in the analyses. The TPA test was carried out. During the analysis, the product was compressed twice to 50% of its original height. The measuring traverse speed was 1 mm s^−1^. The compression test was performed using a cylindrical compression punch with a diameter of 100 mm. To prepare the samples for the test, a 30 × 30 × 10-mm sample was cut out from the middle part of a 10 mm thick slice. The following textural traits were determined in the analysis: hardness [N], springiness [−], cohesiveness [−], and chewiness [N].

### Determination of the color

A 3Color® SF80 spectrophotometer was used for the analysis. Each sample was analyzed in 10 repetitions (light source: D65, observer: 10º, measuring head aperture: 8 mm). The following parameters were measured: L*—lightness, a*—changes in the color from green to red, and b*—changes in the color from blue to yellow (higher values of a* and b* indicate greater intensity of red and yellow, respectively). Additionally, color saturation C* was calculated using the formula: ${C}^{*}=\sqrt{{a}^{*2}+{b}^{*2}}$ , and the total color change *ΔE* was calculated as follows: $\Delta E=\sqrt{{(\Delta L)}^{2}+{(\Delta a)}^{2}+{(\Delta b)}^{2}}$, where Δ*L*, Δ*a*, and Δ*b*—indices of the difference in the color of the surfaces of samples compared with the control bread.

### Sensory analysis

The sunflower cake-supplemented bread was evaluated by a panel of 52 semi-trained team members 24 h after baking. The selected consumers were trained in the descriptive aspects of the analysis. They assessed the color of the crust and crumb, the smell and taste of the bread, and the consistency of the crust and crumb. The results were presented in a 5-point structural scale (1—“dislike very much” and 5—“like very much”). The panelists were selected among the staff and students (aged 21–64) of the University of Life Sciences in Lublin. The following selection criteria were established: good health, non-smoker, and voluntary participation. The test was carried out in a laboratory under LED lighting and at ambient temperature. The panelists were supplied mineral water as a neutralizing agent. The samples were served in random order.

### Statistical analysis

The study results were analyzed using the Statistica 13 software. Numerical values were subjected to one-way analysis of variance (ANOVA). The significance of the differences was verified using Tukey’s test, with the significance level α = 0.05. The results are presented as means ± standard deviation. The sensory evaluation results are presented in a graphical form.

## Supplementary Information

Supplementary Tables.

## Data Availability

All data generated or analysed during this study are included in this published article [and its supplementary information files].
