# Supplementary material for: Sunflower seed cake as a source of nutrients in gluten-free bread
Source: Sci Rep. 2023 Jul 5;13:10864. doi: 10.1038/s41598-023-38094-w (PMC10322822; doi:10.1038/s41598-023-38094-w)
Supplement: Supplementary file 1 — Supplementary Tables. [file 41598_2023_38094_MOESM1_ESM.pdf]

Supplementary file - raw data

Table S1. Physical properties of gluten-free bread with sunflower seed cake

Probe	Repetition number	Crumb moisture	Bread volume	Total baking loss
		[g·100 g^{-1}]	[cm^3]	[g_{loss}·100 g^{-1}]
SC0	1	49,35	1209,6	12,47
SC0	2	49,34	1249,92	12,29
SC0	3	49,37	1310,4	12,49
SC5	1	49,36	1189,44	12,29
SC5	2	49,38	1249,92	12,41
SC5	3	49,36	1229,76	12,37
SC10	1	48,6	1189,44	11,84
SC10	2	48,7	1169,28	12,04
SC10	3	48,62	1189,44	12,12
SC15	1	47,86	1169,28	11,97
SC15	2	47,88	1189,44	11,97
SC15	3	47,87	1189,44	11,98

SC0 – control probe, SC5 – gluten-free bread with 5% sunflower cake added; SC10 - gluten-free bread with 10% sunflower cake added; SC15 - gluten-free bread with 15% sunflower cake added.

Table S2. Chemical compositions of gluten-free bread with sunflower seed cake

Probe	Repetition number	Protein	Fat	Polyphenols	Soluble sugars	Crude fiber
		[$\%_{d.b.}$]	[$\%_{d.b.}$]	[$\%_{d.b.}$]	[mg·100 $g_{d.b.}^{-1}$]	[$\%_{d.b.}$]
SC0	1	7,433	3,35	85,7	2,46	1,21
SC0	2	7,451	3,38	92,8	2,41	1,25
SC0	3	7,441	3,52	89,4	2,39	1,23
SC5	1	8,511	6,5	148,8	2,47	1,91
SC5	2	8,539	6,72	149,3	2,58	1,91
SC5	3	8,513	6,51	148	2,55	1,94
SC10	1	9,291	7,6	189,9	2,61	1,99
SC10	2	9,338	7,89	190,5	2,66	1,98
SC10	3	9,302	7,87	191,4	2,58	2,01
SC15	1	9,644	10,74	222,4	2,62	2,31
SC15	2	9,720	10,71	221,7	2,77	2,35
SC15	3	9,717	10,71	222,9	2,79	2,37

SC0 – control probe, SC5 – gluten-free bread with 5% sunflower cake added; SC10 - gluten-free bread with 10% sunflower cake added; SC15 - gluten-free bread with 15% sunflower cake added.

Table S3. Texture profile analysis of gluten-free bread with sunflower seed cake

**Storage time** [h]	**Repetition number**	**Probe**	**Hardness** [N]	**Elasticity** [-]	**Chewing** [N]	**Cohesiveness** [-]
24	1	SC0	39,3	0,97	6,09936	0,16
24	2	SC0	45,8	0,97	7,10816	0,16
24	3	SC0	42,7	0,96	8,1984	0,2
24	4	SC0	41,7	0,98	7,35588	0,18
24	5	SC0	43,6	0,96	7,11552	0,17
24	1	SC5	30,4	0,87	5,2896	0,2
24	2	SC5	35,1	0,77	5,4054	0,2
24	3	SC5	32,5	0,7	5,005	0,22
24	4	SC5	32,5	0,75	4,63125	0,19
24	5	SC5	35,7	0,87	5,28003	0,17
24	1	SC10	32,1	0,59	4,73475	0,25
24	2	SC10	38,1	0,57	4,77774	0,22
24	3	SC10	33,7	0,65	4,16195	0,19
24	4	SC10	34	0,54	4,59	0,25
24	5	SC10	36,7	0,53	3,50118	0,18
24	1	SC15	22,8	0,58	3,04152	0,23
24	2	SC15	27,1	0,55	4,32245	0,29
24	3	SC15	21,9	0,63	3,03534	0,22
24	4	SC15	27,3	0,52	3,12312	0,22
24	5	SC15	26,7	0,52	2,63796	0,19
48	1	SC0	53,4	0,72	7,30512	0,19
48	2	SC0	68,3	0,73	8,47603	0,17
48	3	SC0	57,2	0,76	8,6944	0,2
48	4	SC0	61,4	0,75	8,7495	0,19
48	5	SC0	56,4	0,73	7,82268	0,19
48	1	SC5	60,5	0,55	8,98425	0,27
48	2	SC5	53,1	0,66	8,06058	0,23
48	3	SC5	58	0,65	9,048	0,24
48	4	SC5	55,4	0,66	8,77536	0,24
48	5	SC5	52,9	0,54	7,99848	0,28
48	1	SC10	39,4	0,56	6,39856	0,29
48	2	SC10	51,3	0,53	7,06914	0,26
48	3	SC10	43,2	0,56	6,048	0,25
48	4	SC10	44,1	0,54	6,42978	0,27
48	5	SC10	40,3	0,56	6,54472	0,29
48	1	SC15	32,6	0,46	4,19888	0,28
48	2	SC15	42,6	0,49	6,47094	0,31
48	3	SC15	38,7	0,46	4,98456	0,28
48	4	SC15	40,5	0,47	5,52015	0,29
48	5	SC15	37,6	0,46	5,36176	0,31

SC0 – control probe, SC5 – gluten-free bread with 5% sunflower cake added; SC10 - gluten-free bread with 10% sunflower cake added; SC15 - gluten-free bread with 15% sunflower cake added

Table S4. Color parameters of gluten-free bread supplemented with sunflower seed cake

**Probe**	**Repetition number**	**L***	**a***	**b***	***C**
SC0	1	62,47	1,27	22,26	22,2962
SC0	2	62,01	1,47	23,5	23,54593
SC0	3	63,36	1,55	22,6	22,65309
SC0	4	62,29	1,72	22,87	22,93459
SC0	5	60,83	1,94	22,43	22,51374
SC0	6	62,11	1,97	23,92	24,00099
SC0	7	60,83	1,91	21,56	21,64444
SC0	8	62,57	1,83	24,37	24,43861
SC0	9	60,57	1,68	23,12	23,18096
SC0	10	61,69	1,84	21,97	22,04692
SC5	1	60,54	1,94	23,26	23,34076
SC5	2	61,26	1,93	24,18	24,2569
SC5	3	60,22	2,18	22,21	22,31673
SC5	4	59,28	2,27	24,67	24,77422
SC5	5	56,79	2,04	24,11	24,19615
SC5	6	58,33	2,98	23,32	23,50963
SC5	7	59,14	2,21	25,1	25,1971
SC5	8	58,13	1,75	23,94	24,00388
SC5	9	60,78	2,26	22,17	22,28489
SC5	10	60,14	2,07	23,52	23,61091
SC10	1	59,89	1,95	23,28	23,36153
SC10	2	58,25	2,36	24,83	24,9419
SC10	3	59,74	2,29	25,32	25,42335
SC10	4	60,17	1,96	25,25	25,32596
SC10	5	59,92	2,28	25,07	25,17346
SC10	6	58,91	2,47	25,88	25,9976
SC10	7	57,39	2,33	24,05	24,1626
SC10	8	59,03	2,51	23,21	23,34533
SC10	9	58,33	2,31	21,47	21,59391
SC10	10	57,92	2,22	23,44	23,54489
SC15	1	56,15	2,28	25,9	26,00016
SC15	2	53,55	2,36	24,81	24,92199
SC15	3	55,99	3,03	25,92	26,0965
SC15	4	57,49	2,98	24,49	24,67064
SC15	5	52,46	2,32	25,12	25,22691
SC15	6	59,79	2,45	23,92	24,04514
SC15	7	53,03	2,38	24,12	24,23714
SC15	8	55,36	2,23	26,62	26,71324
SC15	9	57,11	2,29	25,36	25,46318
SC15	10	52,77	2,44	24,6	24,72071

Table S5. Sensory evaluation of gluten-free bread with the addition of sunflower cake

			SC0			
**Repetition number**	**Color of Bread Crust**	**Color of Bread Crumb**	**Aroma**	**Consistency of Crust**	**Consistency of Crumb**	**Palatability**
1	3	5	3	2	4	3
2	3	2	2	3	2	2
3	4	2	2	3	4	2
4	3	3	1	3	1	2
5	3	4	3	4	2	3
6	4	4	4	3	3	3
7	3	4	3	2	3	3
8	3	4	3	3	4	4
9	4	4	3	3	3	3
10	3	4	4	3	3	5
11	4	4	4	4	3	3
12	4	4	4	4	4	4
13	3	4	3	3	4	4
14	3	5	5	2	4	3
15	3	4	4	3	3	3
16	3	5	4	3	4	3
17	4	4	3	3	5	3
18	3	4	4	4	5	4
19	2	5	5	3	4	4
20	2	5	4	3	4	4
21	4	5	4	3	5	5
22	3	4	4	4	5	5
23	3	5	5	3	5	5
24	3	4	4	2	4	5
25	3	4	4	4	4	4
26	4	4	4	3	4	3
27	3	5	5	4	5	5
28	3	2	4	3	3	4
29	3	4	4	4	4	3
30	2	4	3	4	3	2
31	3	4	4	3	2	3

32	3	3	3	3	4	4
33	4	4	4	4	3	3
34	3	5	4	3	3	3
35	4	4	3	4	4	3
36	4	4	4	5	4	4
37	2	3	3	4	3	3
38	3	3	3	2	3	3
39	4	4	4	3	2	4
40	2	3	5	4	3	3
41	3	3	4	4	3	3
42	4	4	3	3	3	3
43	4	3	3	4	2	3
44	3	3	4	3	2	3
45	4	2	4	3	2	2
46	3	3	5	4	3	3
47	4	2	4	3	2	3
48	3	2	4	4	2	3
49	2	3	3	4	2	3
50	4	2	4	4	2	2
51	3	3	3	4	3	3
52	3	3	4	4	3	3

SC5

**Repetition number**	**Color of Bread Crust**	**Color of Bread Crumb**	**Aroma**	**Consistency of Crust**	**Consistency of Crumb**	**Palatability**
1	4	5	3	3	4	3
2	3	2	2	4	3	2
3	4	2	2	4	4	2
4	4	3	2	3	3	4
5	3	4	4	5	2	4
6	3	4	4	3	3	3
7	4	4	4	4	4	4
8	4	4	4	3	4	4
9	3	4	3	4	4	4
10	4	4	4	5	3	4
11	4	4	4	3	4	4

12	4	4	4	3	4	5
13	4	4	4	4	3	4
14	5	5	5	5	4	4
15	4	5	5	4	4	4
16	4	5	4	4	4	3
17	3	4	3	3	5	4
18	4	4	4	3	4	4
19	3	5	5	3	5	5
20	4	5	4	4	4	4
21	4	5	5	5	5	5
22	3	4	5	4	5	5
23	3	5	5	4	4	5
24	4	4	4	4	4	5
25	4	4	4	4	4	5
26	4	4	4	3	4	4
27	3	5	5	5	5	5
28	3	5	3	4	3	4
29	3	4	4	4	5	4
30	4	4	3	3	4	3
31	4	4	5	4	4	5
32	5	5	4	5	3	4
33	3	3	4	3	5	3
34	4	5	5	5	3	3
35	4	4	3	4	2	4
36	3	5	4	5	4	4
37	4	5	4	4	3	5
38	3	3	4	3	4	3
39	3	4	4	3	4	3
40	5	3	4	4	5	4
41	4	5	4	5	3	3
42	4	3	3	4	4	4
43	4	5	4	4	3	3
44	4	4	3	5	3	4
45	5	5	4	4	3	4
46	4	3	4	3	4	3

47	4	5	4	5	3	3
48	3	5	3	4	4	3
49	3	4	4	4	3	4
50	4	3	4	5	4	4
51	4	3	3	4	3	3
52	4	4	4	4	4	3

SC10

**Repetition number**	**Color of Bread Crust**	**Color of Bread Crumb**	**Aroma**	**Consistency of Crust**	**Consistency of Crumb**	**Palatability**
1	4	5	3	5	4	4
2	5	2	3	5	3	3
3	4	2	2	4	4	2
4	4	4	5	5	4	5
5	4	4	4	4	3	3
6	4	4	4	5	4	4
7	5	4	4	4	4	3
8	4	4	4	5	4	4
9	5	3	2	5	3	2
10	4	4	3	5	4	5
11	4	3	3	4	2	2
12	4	4	4	4	4	4
13	4	4	3	5	3	4
14	4	5	5	4	4	3
15	5	4	5	5	4	4
16	5	5	4	5	4	5
17	5	4	3	5	5	1
18	4	5	5	5	5	4
19	5	5	5	4	4	5
20	4	5	5	4	5	5
21	5	5	5	5	5	4
22	5	5	5	4	5	4
23	4	5	5	5	5	5
24	4	4	4	4	4	5
25	4	4	4	5	4	3
26	3	5	4	4	5	5

27	5	5	5	5	5	5
28	3	4	4	4	4	5
29	4	3	3	5	5	5
30	4	5	3	5	5	4
31	5	4	5	5	4	4
32	4	5	4	5	5	5
33	3	5	3	4	4	3
34	5	4	4	4	3	5
35	5	4	3	5	4	5
36	5	4	4	5	3	4
37	3	3	4	5	5	4
38	4	4	5	5	5	5
39	3	3	3	5	4	4
40	5	5	4	4	4	5
41	5	4	5	5	5	5
42	4	3	4	5	5	5
43	4	4	4	5	5	5
44	4	3	4	5	4	5
45	5	4	3	4	5	4
46	4	4	4	5	4	5
47	4	5	4	4	5	4
48	4	4	5	4	4	5
49	4	4	3	5	4	5
50	3	4	4	5	5	5
51	4	5	3	5	4	5
52	4	4	3	4	5	5

SC15

**Repetition number**	**Color of Bread Crust**	**Color of Bread Crumb**	**Aroma**	**Consistency of Crust**	**Consistency of Crumb**	**Palatability**
1	5	5	3	5	4	4
2	5	2	3	4	4	5
3	4	2	2	4	4	5
4	5	3	3	4	3	4
5	4	3	4	4	3	4
6	4	4	5	4	5	5

7	4	4	3	5	4	3
8	5	4	4	4	4	4
9	4	2	3	4	4	4
10	5	5	4	4	5	5
11	4	4	4	4	4	4
12	4	5	5	4	5	4
13	5	4	4	5	4	4
14	5	5	5	4	4	3
15	4	5	5	4	5	5
16	5	5	5	4	5	5
17	4	4	3	4	5	2
18	5	5	5	4	4	3
19	5	5	5	5	4	3
20	5	5	5	4	5	5
21	5	5	5	4	5	4
22	4	5	5	4	5	5
23	4	5	5	5	4	4
24	4	4	4	4	4	4
25	4	4	4	4	4	4
26	4	5	4	4	5	5
27	5	5	5	5	5	5
28	4	5	4	4	5	4
29	4	4	4	5	5	5
30	5	5	4	5	4	5
31	5	4	5	4	5	5
32	5	4	4	3	4	4
33	4	5	3	4	3	5
34	5	4	4	5	5	4
35	4	4	4	5	5	3
36	5	5	3	5	4	5
37	5	4	4	4	4	5
38	5	4	4	5	5	4
39	5	5	3	4	4	5
40	4	4	4	5	5	5
41	5	4	5	5	5	5

42	5	5	3	5	5	5
43	5	5	4	4	5	5
44	5	4	3	5	4	5
45	4	4	3	4	5	5
46	5	5	4	4	4	5
47	5	5	3	5	5	5
48	5	5	4	5	4	4
49	5	4	4	4	5	5
50	5	4	3	5	5	5
51	5	4	4	5	4	5
52	4	4	3	4	5	5

SC0 – control probe, SC5 – gluten-free bread with 5% sunflower cake added; SC10 - gluten-free bread with 10% sunflower cake added; SC15 - gluten-free bread with 15% sunflower cake added

The results were presented in a 5-point structural scale (from 1 -“dislike very much” to 5 - “like very much”)
